# Supplementary figures and images for: Benefit finding and well-being over the course of the COVID-19 pandemic
Source: PLoS One. 2023 Jul 27;18(7):e0288332. doi: 10.1371/journal.pone.0288332 (PMC10374125; doi:10.1371/journal.pone.0288332)

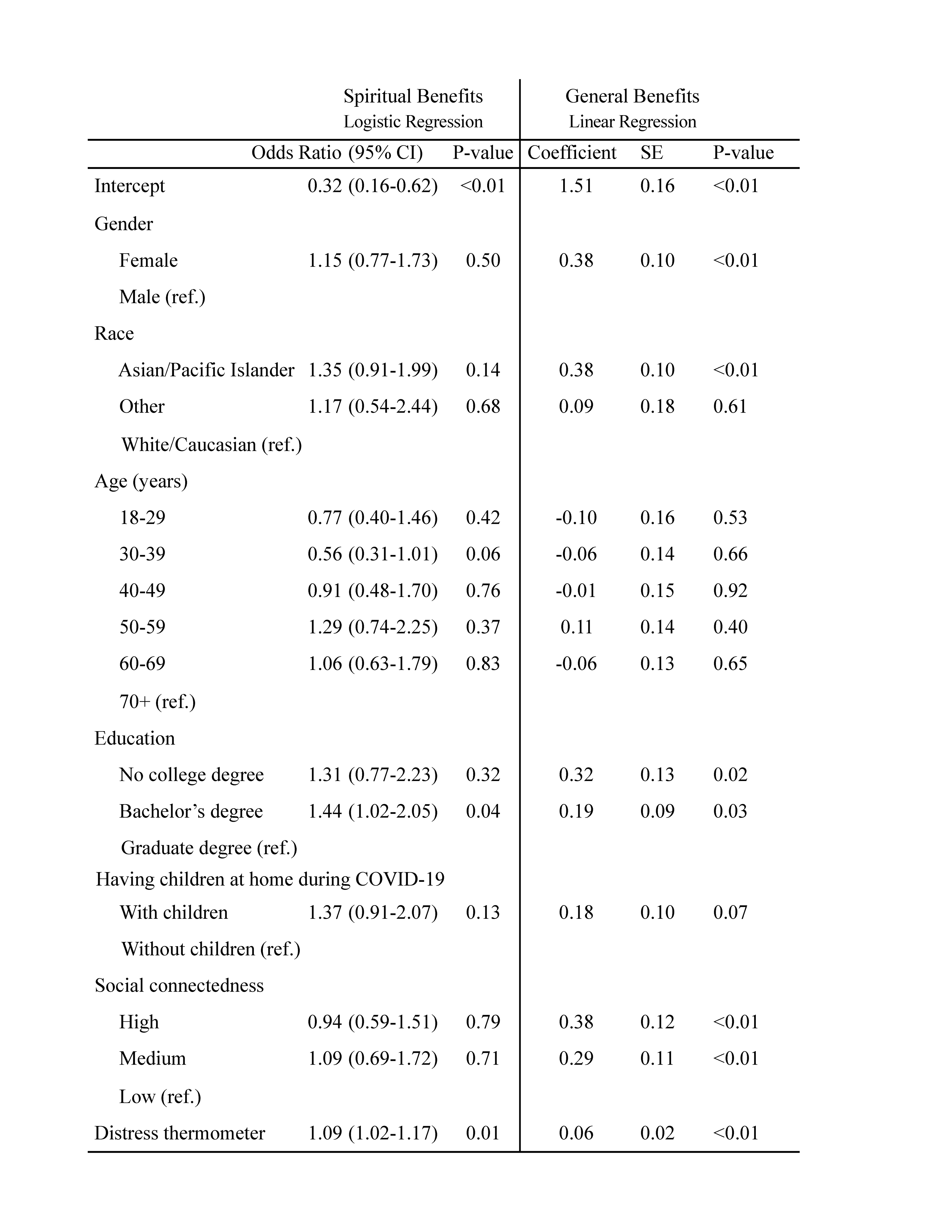

Supplement: S1 Table — (TIF) [file pone.0288332.s001.tif]

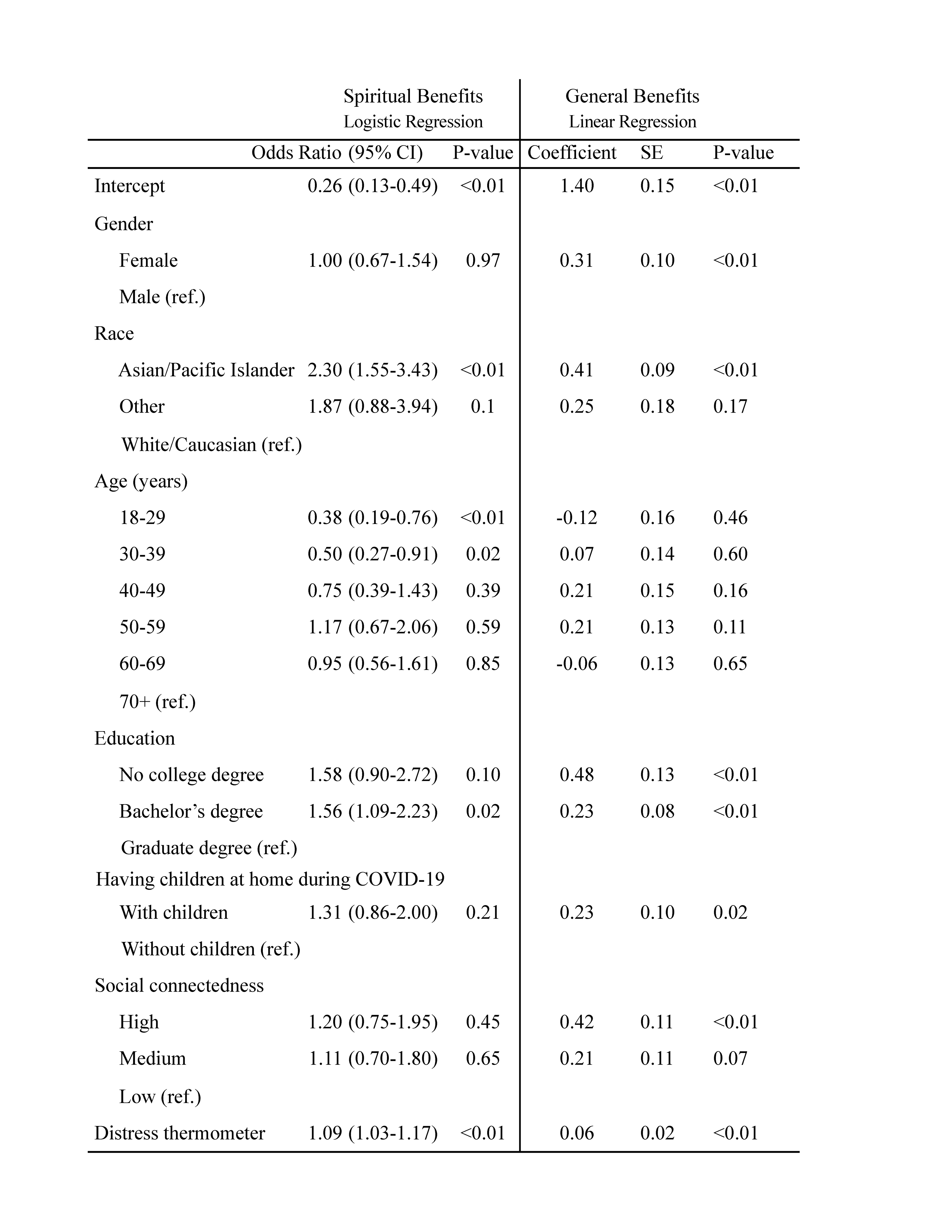

Supplement: S2 Table — (TIF) [file pone.0288332.s002.tif]

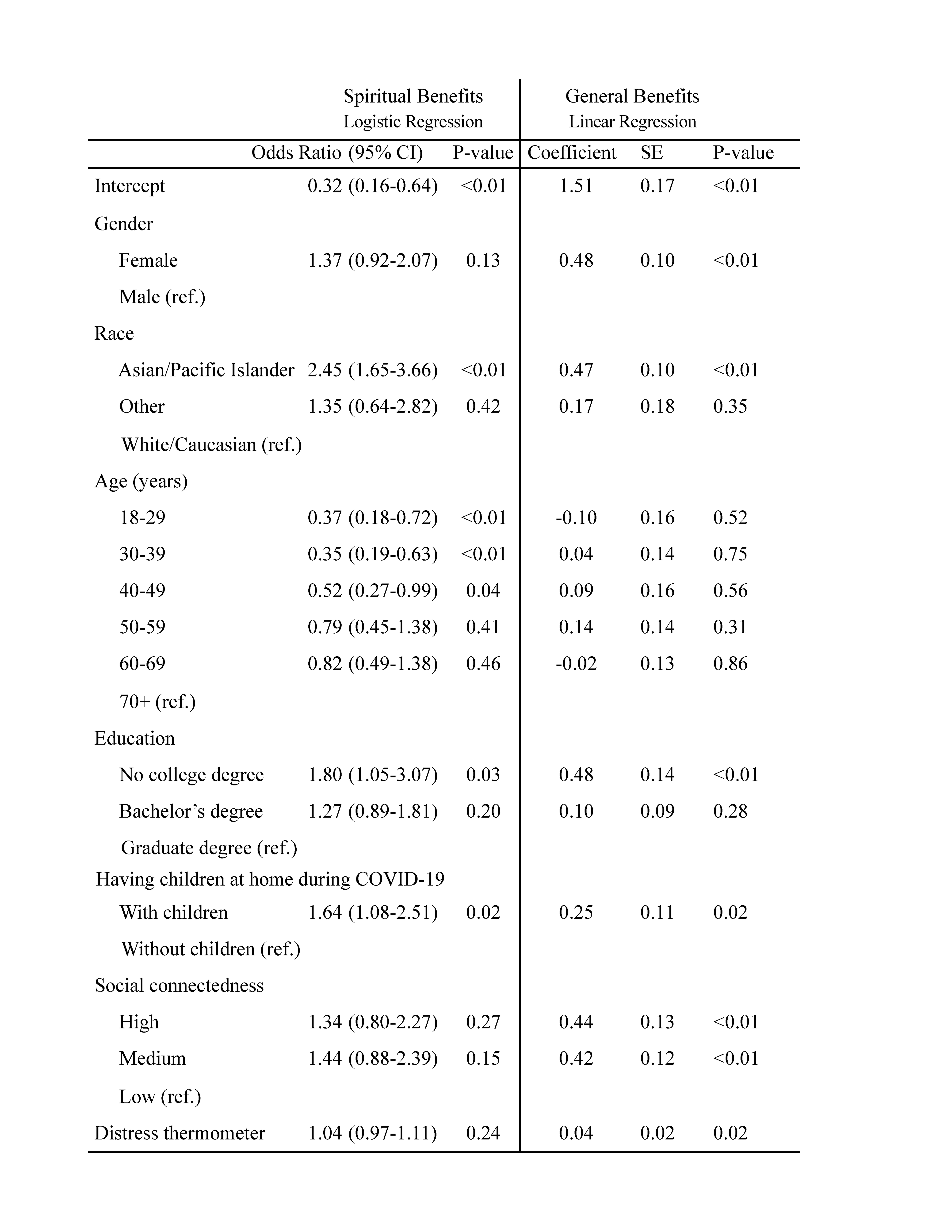

Supplement: S3 Table — (TIF) [file pone.0288332.s003.tif]

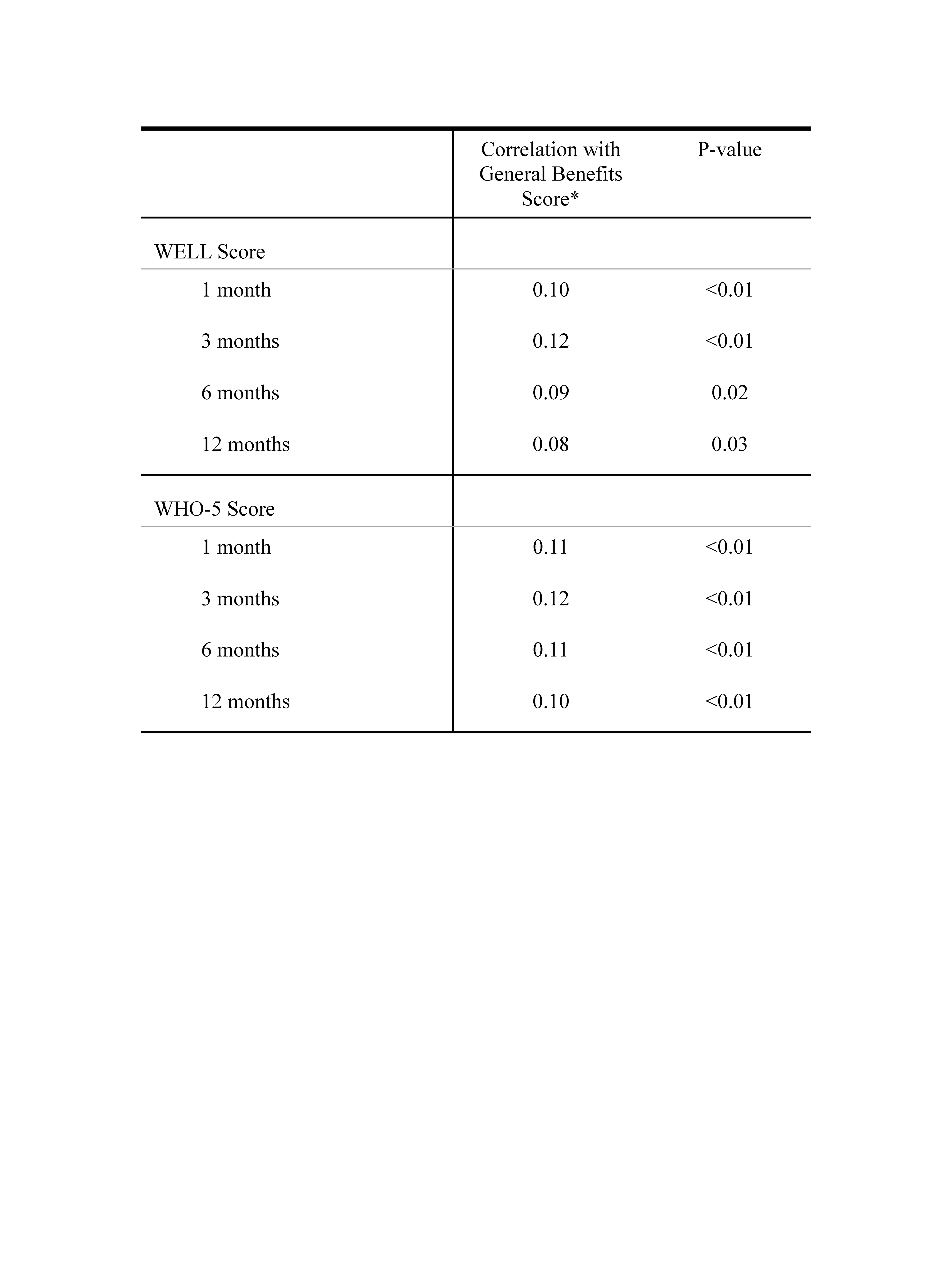

Supplement: S4 Table — *The General Benefits Score at 1 month, 3 months, 6 months, and 12 months was used, respectively. (TIF) [file pone.0288332.s004.tif]
